# Extent of the Acidification by N7-Coordinated *cis*-Diammine-Platinum(II) on the Acidic Sites of Guanine Derivatives

**DOI:** 10.1155/MBD.1996.131

**Published:** 1996

**Authors:** Bin Song, Gerda Oswald, Matthias Bastian, Helmut Sigel, Bernhard Lippert

**Affiliations:** 1 Institute of Inorganic Chemistry University of Basel Spitalstrasse 51 Basel CH-4056 Switzerland; 2 Department of Chemistry University of Dortmund Otto-Hahn-Strasse 6 Dortmund D-44221 Germany

## Abstract

Coordination of two monoprotonated 2'-deoxyguanosine 5'-monophosphate species, H(dGMP)^−^, via N7 to *cis*-(NH_2_)_2_Pt^2+^ gives the complex *cis*-(NH_2_)_2_Pt(H·dGMP)_2_ which is a four-protonic acid. The corresponding acidity constants were measured by potentiometric pH titrations (25℃; *I* = 0.1 M, NaNO_3_). The first two protons are released from the two -P(O)_2_(OH)^−^ groups (P*K*_a/1_= 5.57; P*K*_a/2_ = 6.29) and the next two protons are from the H(N1) sites of the guanine residues (P*K*_a/3_ = 8.73; P*K*_a/4_ = 9.48). The micro acidity constants of the various sites are also evaluated. Comparison of these data with those determined for the three-protonic H_2_(dGMP)^±^ (P*K*_a/1_ = 2.69 for the H^+^(N7) site; P*K*_a/2_ = 6.29 for -P(O)_2_(OH)^−^ ;P*K*_a/3_ = 9.56 for H(N1)) shows that on average the N-7-coordinated Pt^2+^ acidifies the phosphate protons by Δ p*K*_a_ = 0.36 and the H(N1) sites by Δ p*K*_a_ = 0.46. These results are further compared with those obtained previously for *cis*-(NH_2_)_2_Pt(L)_2_, where L = 9-ethylguanine or monoprotonated 2'-deoxycytidine 5'-monophosphate. Conclusions regarding platinated DNA are also presented.


### EXTENT OF THE ACIDIFICATION BY N7-COORDINATED *cis*-DIAMMINE-PLATINUM(II) ON THE ACIDIC SITES OF GUANINE DERIVATIVES

Bin Song[1], Gerda Oswald[2], Matthias Bastian[1], Helmut Sigel*[1] and Bernhard Lippert*[2]

[1] Institute of Inorganic Chemistry, University of Basel, Spitalstrasse 51, CH-4056 Basel, Switzerland

[2] Department of Chemistry, University of Dortmund, Otto-Hahn-Strasse 6, D-44221 Dortmund, Germany

### Abstract

Coordination of two monoprotonated 2'-deoxyguanosine 5'-monophosphate species, $H(dGMP)^-$, via N7 to *cis*-$(NH_3)_2Pt^{2+}$ gives the complex *cis*-$(NH_3)_2Pt(H{\cdot}dGMP)_2$ which is a four-protonic acid. The corresponding acidity constants were measured by potentiometric pH titrations (25°C; $I$ = 0.1 M, $NaNO_3$). The first two protons are released from the two $-P(O)_2(OH)^-$ groups ($pK_{a/1}$ = 5.57; $pK_{a/2}$ = 6.29) and the next two protons are from the H(N1) sites of the guanine residues ($pK_{a/3}$ = 8.73; $pK_{a/4}$ = 9.48). The micro acidity constants of the various sites are also evaluated. Comparison of these data with those determined for the three-protonic $H_2(dGMP)^{\pm}$ ($pK_{a/1}$ = 2.69 for the $H^+$(N7) site; $pK_{a/2}$ = 6.29 for $-P(O)_2(OH)^-$; $pK_{a/3}$ = 9.56 for H(N1)) shows that on average the N-7-coordinated $Pt^{2+}$ acidifies the phosphate protons by $\Delta$ $pK_a$ = 0.36 and the H(N1) sites by $\Delta$ $pK_a$ = 0.46. These results are further compared with those obtained previously for *cis*-$(NH_3)_2Pt(L)_2$, where L = 9-ethylguanine or monoprotonated 2'-deoxycytidine 5'-monophosphate. Conclusions regarding platinated DNA are also presented.

### 1. INTRODUCTION

There is now much evidence that the anticancer drug, *Cisplatin*,† i.e. *cis*-diammine-dichloro-platinum(II), loses in the cell the chloro ligands and exerts then its biological action

---

†**Abbreviations:** *cis*-$(NH_3)_2Pt^{2+}$ = *cis*-diammine-platinum(II); *cis*-$(NH_3)_2Pt(dCMP)_2^{2-}$ = $Pt(dCMP)_2^{2-}$; *cis*-$(NH_3)_2Pt(dGMP)_2^{2-}$ = $Pt(dGMP)_2^{2-}$; *cis*-$(NH_3)_2Pt(9\text{-}EtG)_2^{2+}$ = $Pt(9\text{-}EtG)_2^{2+}$; $dCMP^{2-}$ = 2'-deoxy-cytidine 5'-monophosphate; $dGMP^{2-}$ = 2'-deoxyguanosine 5'-monophosphate; $(dGMP{-}H)^{3-}$ = N1-deprotonated $dGMP^{2-}$; 9-EtG = 9-ethylguanine; $(9\text{-}EtG{-}H)^-$ = N1-deprotonated 9-EtG; L = general ligand. Species which are given without a charge either do not carry one or represent the species in general (i.e., independent from their protonation degree); which of the two versions applies is always clear from the context.

by the preferred binding of *cis*-$(NH_3)_2Pt^{2+}$ to the N7 sites of the guanine residues of DNA.[1,2] Consequently, the coordination chemistry of *cis*-diammine-platinum(II) has much been studied, especially its interaction with nucleobases (e.g.[3,4]), as have the metal ion-binding properties of nucleobase derivatives in general (e.g.[5,6]).

However, so far there is no comprehensive study which examines the effect of N7-coordinated *cis*-$(NH_3)_2Pt^{2+}$ on the acid-base properties of guanine derivatives. We have recently published some data[7] on *cis*-$(NH_3)_2Pt(9\text{-}EtG)_2^{2+}$ and a preliminary abstract[8] dealing with *cis*-$(NH_3)_2Pt(dGMP)_2^{2-}$. The latter study has been completed in the meantime and therefore we are now in the position to compare the effect of N7-coordinated *cis*-$(NH_3)_2Pt^{2+}$ on the N1 sites of 9-EtG with that on the corresponding sites of $dGMP^{2-}$ (Figure 1) as well as on the monoprotonated phosphate groups in *cis*-$(NH_3)_2Pt(H{\cdot}dGMP)_2$. The latter effect may further be compared with the situation of monoprotonated 2'-deoxycytidine 5'-monophosphate, $H(dCMP)^-$, if bound via N3 to *cis*-$(NH_3)_2Pt^{2+}$; this complex, i.e. *cis*-$(NH_3)_2Pt(H{\cdot}dCMP)_2$ (see Figure 2, vide infra), has also been studied.[9]

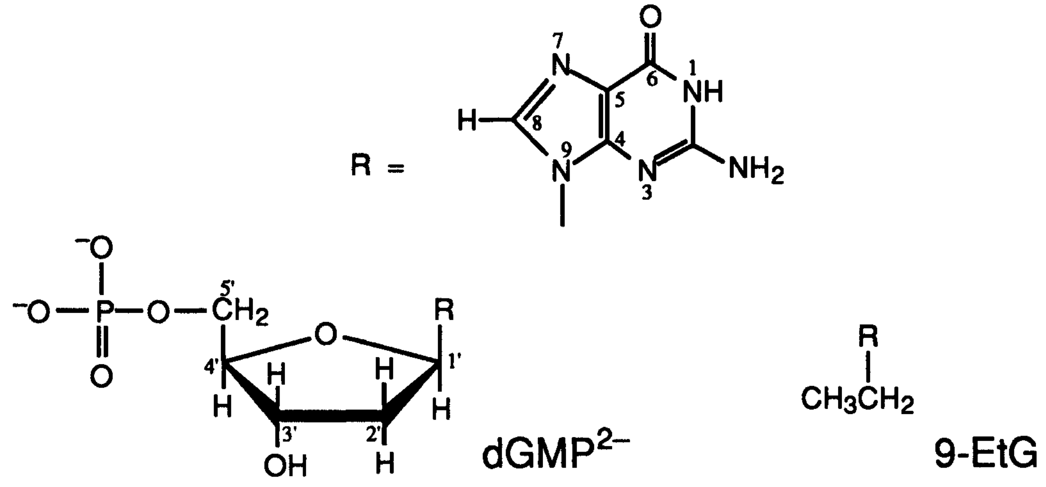

**Figure 1.** Chemical structures of 2'-deoxyguanosine 5'-monophosphate ($dGMP^{2-}$) and of 9-ethylguanine (9-EtG).

### 2. MATERIALS AND METHODS

### *2.1. Synthesis of* cis-$(NH_3)_2Pt(dGMP)_2^{2-}$

To a solution of $H_2(dGMP){\cdot}2\,H_2O$ (0.53 mmol, 202 mg) in water (50 mL) is added *cis*-$(NH_3)_2PtCl_2$ (0.28 mmol, 84 mg) and the pH is adjusted by means of NaOH to 7.3. After stirring for 5 days (room temperature, stoppered flask) the sample is filtered from a small amount of grey precipitate (presumably elemental Pt) and brought to dryness by rotary evaporation at 40°C. The crude product is passed over a Sephadex G10 column (FPLC,

Pharmacia/LBK) to remove NaCl and then brought to dryness at 40°C. Anal. calcd (found) for $Na_2[(NH_3)_2Pt(dGMP)_2]\cdot 11\ H_2O$ (**1**): C, 20.6 (21.2); H, 4.5 (4.0); N, 14.4 (14.0). The yield was 204 mg (66%).

Addition of HCl to an aqueous solution of **1** (0.09 mmol, 100 mg; 3 mL $H_2O$; pH 1.6) and slow evaporation in air yields within several days 40 mg (43%) of colorless crystals of *cis*-$(NH_3)_2Pt(H\cdot dGMP)_2\cdot 6.5\ H_2O$ (**2**). Anal. calcd (found): C, 23.1 (23.1); H, 4.4 (4.4); N, 16.2 (16.5). Thermogravimetry is consistent with 6.5 $H_2O$ molecules present.

Coordination of the two dGMPs via N7 to *cis*-$(NH_3)_2Pt^{2+}$ is confirmed by the acid-base properties of **1** described in this study; these are in accord with a $Pt^{2+}$-N7 coordination in *cis*-$(NH_3)_2Pt(H\cdot dGMP)_2$ only.

### 2.2. Materials and Apparatus for the Titration Experiments

The disodium salt of 2'-deoxyguanosine 5'-monophosphate was purchased from Sigma Chemical Co., St. Louis, MO, USA. Potassium hydrogen phthalate, $NaNO_3$, $HNO_3$ and NaOH (Titrisol) (all *pro analysi*) were obtained from Merck AG, Darmstadt, Germany. The disodium salt of $Pt(dGMP)_2^{2-}$ was prepared as described in Section 2.1. For all solutions distilled $CO_2$-free water was used.

The titer of the NaOH used for the titrations was determined with potassium hydrogen phthalate. The stock solutions of $dGMP^{2-}$ and *cis*-$(NH_3)_2Pt(dGMP)_2^{2-}$ were freshly prepared daily, and the pH was adjusted to about 8.4 and 7.6, respectively; the exact concentrations of these solutions (titrated in the presence of an excess of $HNO_3$; see Section 2.3) were measured by titrations with NaOH.

The potentiometric pH titrations were carried out with a Metrohm E536 potentiograph equipped with an E665 dosimat and a 6.0202.100(NB) combined macro glass electrode. The buffer solutions (pH 4.64, 7.00, 9.00; based on the NIST scale) used for calibration were also from Metrohm AG, Herisau, Switzerland. The direct pH meter readings were used in the calculations of the acidity constants; i.e., these constants are so-called practical, mixed or Brønsted constants.[10] Their negative logarithms given for aqueous solutions at $I$ = 0.1 M and 25°C may be converted into the corresponding concentration constants by subtracting 0.02 from the listed p$K_a$ values.[9,10]

### 2.3. Potentiometric pH Titrations

The acidity constants $K^H_{H_2(dGMP)}$, $K^H_{H(dGMP)}$ and $K^H_{dGMP}$ of $H_2(dGMP)^{\pm}$ were determined by titrating 50 mL of aqueous 1.08 mM $HNO_3$ (25°C; $I$ = 0.1 M, $NaNO_3$) in the presence and absence of 0.3 mM or 0.4 mM of dGMP under $N_2$ with 3 mL 0.03 M or 2 mL

0.045 M NaOH, respectively, and by using the differences in NaOH consumption between two such titrations for the calculations. The constants were calculated with an IBM compatible computer with an 80486 processor (connected with a Brother M1509 printer and a Hewlett-Packard 7475A plotter) by a curve-fit procedure using a Newton-Gauss non-linear least-squares program within the pH range 3.1 to 10.3, corresponding to about 72% neutralisation for the equilibrium $H_2(dGMP)^{\pm}/H(dGMP)^-$ and about 85% neutralisation for the equilibrium $dGMP^{2-}/(dGMP{-}H)^{3-}$. The results listed in Table 1 are the averages of 18 independent pairs of titrations.

The acidity constants $K^H_{Pt(H \cdot dGMP)_2}$, $K^H_{Pt(dGMP)(H \cdot dGMP)}$, $K^H_{Pt(dGMP)_2}$ and $K^H_{Pt(dGMP-H)(dGMP)}$ of *cis*-$(NH_3)_2Pt(H \cdot dGMP)_2$, abbreviated as $Pt(H \cdot dGMP)_2$, were determined by titrating 25 mL of aqueous 1.08 mM $HNO_3$ (25°C; $I$ = 0.08-0.1 M, $NaNO_3$) in the presence and absence of 0.4 mM $Pt(dGMP)_2$ under $N_2$ with 2 mL of 0.03 M NaOH and by using the differences in NaOH consumption between two such titrations for the calculations. These calculations were carried out as indicated above within the pH range 3.6 to 10.3, corresponding to about 1% neutralisation for the equilibrium $Pt(H \cdot dGMP)_2/Pt(dGMP)(H \cdot dGMP)^-$ and about 87% neutralisation for the equilibrium $Pt(dGMP{-}H)(dGMP)^{3-}/Pt(dGMP{-}H)_2^{4-}$. The final results given in Table 1 are the averages of 4 independent pairs of titrations.

### 3. RESULTS AND DISCUSSION

### *3.1. Definition of the Acidity Constants and Results*

A species with a nucleobase residue is always defined as L; hence, such a species may be *mono*-protonated, e.g. at the phosphate group as in $dGMP^{2-}$ or at the N7 site as in 9-EtG, giving H(L), or it may be *de*-protonated, e.g. at H(N1) as in $dGMP^{2-}$, giving (L–H). Therefore, the acidity constants are defined, e.g., according to equilibria (1) to (3); for the sake of clarity the charges are omitted at the ligand species:

$$H_2(L) \rightleftharpoons H(L) + H^+ \quad (1a)$$

$$K^H_{H_2(L)} = [H(L)][H^+]/[H_2(L)] \quad (1b)$$

$$H(L) \rightleftharpoons L + H^+ \quad (2a)$$

$$K^H_{H(L)} = [L][H^+]/[H(L)] \quad (2b)$$

$$L \rightleftharpoons (L{-}H) + H^+ \quad (3a)$$

$$K^H_L = [(L{-}H)][H^+]/[L] \quad (3b)$$

Hence, e.g., $H_2(dGMP)^{\pm}$ (= $H_2(L)$) is deprotonated at $H^+$(N7) (see Figure 1) according to equilibrium (1) giving $H(dGMP)^-$ (= H(L)) which loses next (eq (2)) the proton from the phosphate group giving $dGMP^{2-}$ (= L); this species may further be deprotonated at its H(N1) site to give $(dGMP-H)^{3-}$ (= (L–H); eq (3)). Or, to give a further example: coordination of two $H(dGMP)^-$ species via N7 to *cis*-$(NH_3)_2Pt^{2+}$ results in *cis*-$(NH_3)_2Pt(H{\cdot}dGMP)_2$, a complex which first loses in successive steps two protons from its phosphate residues (cf. also Figure 2, vide infra) according to equilibria (1) and (2); next, one of the two H(N1) sites of the guanine residues (see Figure 1) is ionized to give $Pt(dGMP-H)(dGMP)^{3-}$ according to equilibrium (3) and then in a final step $Pt(dGMP-H)_2^{4-}$ is formed.

The corresponding acidity constants obtained via potentiometric pH titrations for the deprotonation of $H(9\text{-}EtG)^+$ and $H_2(dGMP)^{\pm}$ as well as for their complexes, *cis*-$(NH_3)_2Pt(9\text{-}EtG)_2^{2+}$ and *cis*-$(NH_3)_2Pt(H{\cdot}dGMP)_2$, are listed in Table 1, where the various deprotonation sites are also defined. The constants due to $H(dCMP)^-$ and *cis*-$(NH_3)_2Pt(H{\cdot}dCMP)_2$ are given for comparison.

From the results summarized in Table 1 it is immediately evident that deprotonation of the $-P(O)_2(OH)^-$ residues occurs in all species relatively close to pH 6, whereas the proton from the H(N1) site of the guanine moieties is released in the pH range of about 9. Of some surprise may appear the fact that the $H^+$(N7) site of the positively charged $H(9\text{-}EtG)^+$ re-

**Table 1.** Negative Logrithms of Acidity Constants[a] of Free and Pt-Coordinated Guanine Derivatives as Determined by Potentiometric pH Titrations in Aqueous Solution at 25°C and $I$ = 0.1 M ($NaNO_3$) Together with Some Related Data Determined Under the Same Conditions for Cytidine Species

| H₂(L)/H(L)/L | $pK^H_{H_2(L)}/pK^H_{H(L)}$ $H^+$(N7) | $pK^H_{H_2(L)}$ $-P(O)_2(OH)^-$ | $pK^H_{H(L)}$ $-P(O)_2(OH)^-$ | $pK^H_L$ H(N1) | $pK^H_{(L-H)}$ H(N1) |
|---|---|---|---|---|---|
| $H(9\text{-}EtG)^+$ [7] | 3.27±0.04 | | | 9.57±0.04 | |
| $Pt(9\text{-}EtG)_2^{2+}$ [7] | | | | 8.02±0.01 | 8.67±0.01 |
| $H_2(dGMP)^{\pm}$ | 2.69±0.03 | | 6.29±0.01 | 9.56±0.02 | |
| $Pt(H{\cdot}dGMP)_2$ | | 5.57±0.03 | 6.29±0.02 | 8.73±0.04 | 9.48±0.04 |
| $H(dCMP)^-$ [9][b] | | | 6.24±0.01 | | |
| $Pt(H{\cdot}dCMP)_2$ [9] | | 5.73±0.02 | 6.47±0.02 | | |

[a] The error limits given are *three times* the standard error of the mean value or the sum of the probable systematic errors, whichever is larger.

[b] For the deprotonation of the $H^+$(N3) site holds: $pK^H_{H_2(dCMP)} = 4.46 \pm 0.01$.[9]

leases its proton with p$K_a$ = 3.27 only, whereas from the overall neutral (i.e. zwitter ionic) $H_2(dGMP)^{\pm}$ species the release occurs already with p$K_a$ = 2.69 (Table 1). This lower basicity of N7 in $H(dGMP)^-$ compared with that of 9-EtG is clearly attributable to the sugar residue, and is thus probably a solvation effect because $H(guanosine)^+$ and H(2'-deoxy-guanosine)$^+$ are $H^+$(N7)-deprotonated with p$K_a$ = 2.11 ± 0.04[11] and p$K_a$ = 2.30 ± 0.04,[12] respectively. Comparison of the values for $H(dGuo)^+$ (p$K_a$ = 2.30) and $H(d{\cdot}GMP)^-$ (p$K_a$ = 2.69) shows that the expected charge effect is now operating.

### *3.2. Some Statistical Considerations on the cis-$(NH_3)_2Pt(L)_2$ Species*

From Figure 2, where a simplified structure of *cis*-$(NH_3)_2Pt(H{\cdot}dCMP)_2$ is shown, it is immediately obvious that this complex as well as *cis*-$(NH_3)_2Pt(9\text{-}EtG)_2^{2+}$ or *cis*-$(NH_3)_2Pt(H{\cdot}dGMP)_2$ are 'symmetrical' di- (and tetra-) protonic acids, just like dihydrogen sulfide, $H_2S$, or oxalic acid, HO(O)C-C(O)OH. The statistical expectation for the separation of the acidity constants of two identical acidic sites in the same molecule, which do *not* affect each other, is Δ p$K_{a/st}$ = 0.6.[9] This follows from the symmetry properties of, e.g., *cis*-$(NH_3)_2Pt(dCMP)_2$: Beginning with $Pt(H{\cdot}dCMP)_2$ there are two equivalent ways (see Figure 2) to form $Pt(dCMP)(H{\cdot}dCMP)^-$; on the other hand, there are also two equivalent ways for the protonation of $Pt(dCMP)_2^{2-}$ to give $Pt(dCMP)(H{\cdot}dCMP)^-$. This means, the

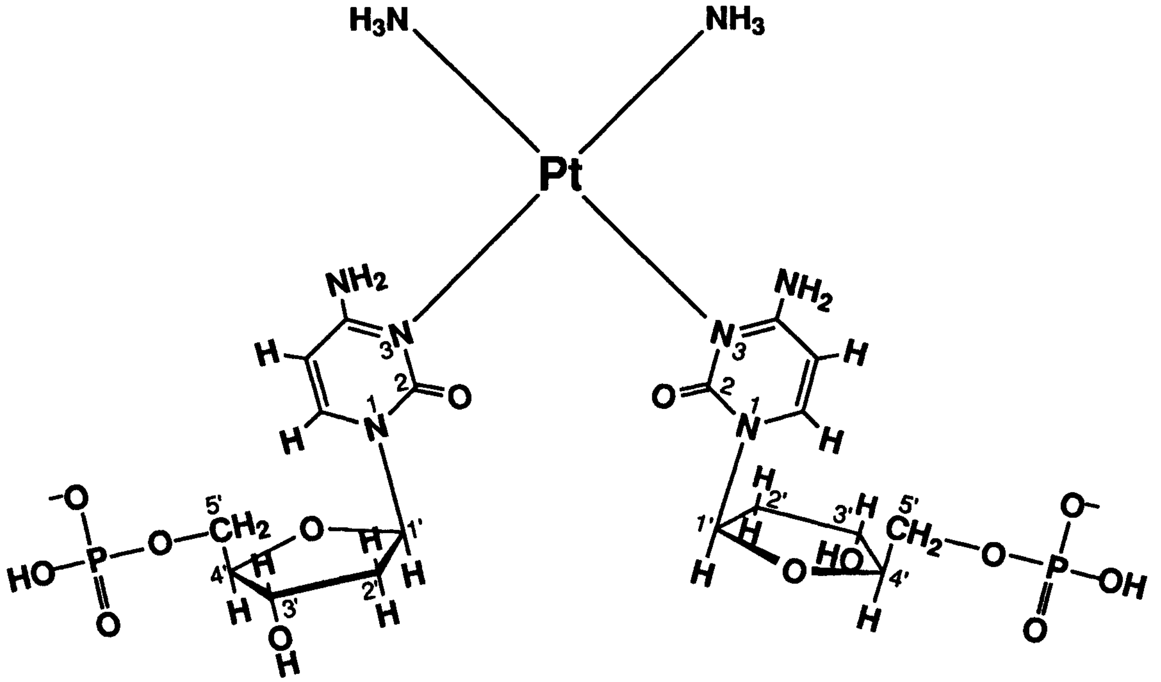

**Figure 2.** A possible and simplified structure of the *cis*-$(NH_3)_2Pt(H{\cdot}dCMP)_2$ complex showing the two monoprotonated $-P(O)_2(OH)^-$ groups.

formation of the monoprotonated species $Pt(dCMP)(H{\cdot}dCMP)^-$ is two times favored by a factor of 2 which gives overall a factor of 4, i.e. $\Delta\, pK_{a/st} = 0.6$.

The above value has to be compared with the following ones:

$$\begin{aligned}\Delta\, pK_{a/Pt,dCMP} &= pK^H_{Pt(dCMP)(H\cdot dCMP)} - pK^H_{Pt(H\cdot dCMP)_2}\\ &= (6.47 \pm 0.02) - (5.73 \pm 0.02)\\ &= 0.74 \pm 0.03 \qquad (4)\end{aligned}$$

$$\begin{aligned}\Delta\, pK_{a/Pt,9\text{-}EtG} &= pK^H_{Pt(9\text{-}EtG-H)(9\text{-}EtG)} - pK^H_{Pt(9\text{-}EtG)_2}\\ &= (8.67 \pm 0.01) - (8.02 \pm 0.01)\\ &= 0.65 \pm 0.01 \qquad (5)\end{aligned}$$

$$\begin{aligned}\Delta\, pK_{a/Pt,H\cdot dGMP} &= pK^H_{Pt(dGMP)(H\cdot dGMP)} - pK^H_{Pt(H\cdot dGMP)_2}\\ &= (6.29 \pm 0.02) - (5.57 \pm 0.03)\\ &= 0.72 \pm 0.04 \qquad (6)\end{aligned}$$

$$\begin{aligned}\Delta\, pK_{a/Pt,dGMP} &= pK^H_{Pt(dGMP-H)(dGMP)} - pK^H_{Pt(dGMP)_2}\\ &= (9.48 \pm 0.04) - (8.73 \pm 0.04)\\ &= 0.75 \pm 0.06 \qquad (7)\end{aligned}$$

It becomes thus evident that all these $\Delta\, pK_a$ values are close to the statistical expectation; they are on average only about 0.1 $pK$ units larger than $\Delta\, pK_{a/st} = 0.6$. In other words, the mutual influence that the two corresponding acidic sites in these complexes exert on each other is quite small, which indicates that the distances between these sites (at least in the protonated forms) must be relatively large.

### *3.3. Micro Acidity Constants for the* cis-$(NH_3)_2Pt(L)_2$ *Species and Acidifying Effect of the N7-Coordinated* $Pt^{2+}$

The negative logarithms of the two acidity constants, e.g., $pK^H_{Pt(H\cdot dCMP)_2}$ and $pK^H_{Pt(dCMP)(H\cdot dCMP)}$, are only slightly more apart from each other than the statistically expected 0.6 $pK$ units; this means, the buffer regions of the two species, $Pt(H{\cdot}dCMP)_2$ and $Pt(dCMP)(H{\cdot}dCMP)^-$, are strongly overlapping. The same also applies to the other $Pt^{2+}$ complexes considered here; i.e., those formed between *cis*-$(NH_3)_2Pt^{2+}$ and 9-EtG, $H(dGMP)^-$, or $dGMP^{2-}$ (see Table 1). Therefore, for a clean quantification of the acidity of the various sites it is necessary to calculate the micro acidity constants for the individual sites. Following known routes[13,14] we have summarized in Figure 3, as an example, the equilibrium scheme for *cis*-$(NH_3)_2Pt(H{\cdot}dCMP)_2$ defining the micro acidity constants ($k$) and giving their interrelation with the macro acidity constants ($K$). There are three independent equations (a), (b), and (c), but four unknown constants;[13] however, by taking into account the statistical considerations of Section 3.2 the matter becomes simple in the present case because $pK^H_{Pt(H\cdot dCMP)} + \log 2 = 5.73 + 0.3 = 6.03 = pk^1 = pk_1$; the analogues reasoning provides $pk_2$, etc.

The values for the micro acidity constants, $pk^1 = pk_1$ and $pk_2 = pk^2$, for the four *cis*-$(NH_3)_2Pt(L)_2$ complexes appearing in Table 1 are summarized in columns 2 and 3 of Table 2, respectively. Columns 4 and 5 provide the differences between the $pK_a$ values of the free ligands, like 9-EtG or $H(dGMP)^-$, and the values given for $pk^1 = pk_1$ and $pk_2 = pk^2$. Thus, these values quantify the acidifying effect of $Pt^{2+}$ on the individual sites.

For a general comparison, however, we feel it is more appropriate to take the average of the effect that the two 'symmetrical' sites experience by $Pt^{2+}$; e.g., the hydrogens in the

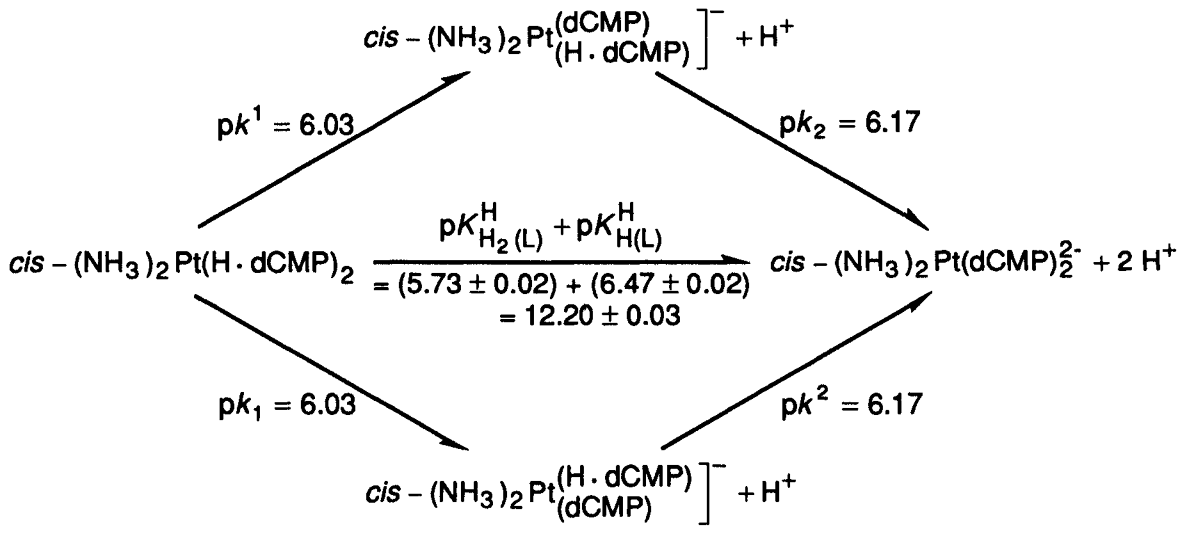

(a) $K^H_{H_2(L)} = k^1 + k_1$

(b) $\frac{1}{K^H_{H(L)}} = \frac{1}{k_2} + \frac{1}{k^2}$

(c) $K^H_{H_2(L)} \cdot K^H_{H(L)} = k^1 \cdot k_2 = k_1 \cdot k^2$

**Figure 3**. Equilibrium scheme for *cis*-$(NH_3)_2Pt(H\cdot dCMP)_2$ defining the micro acidity constants ($k$) and showing their interrelation with the macro acidity constants ($K$). The arrows indicate the direction for which the acidity constants are defined. Equations (a), (b), and (c) show how the various constants are interlinked with each other.[13] See also text in Section 3.3.

two $-P(O)_2(OH)^-$ groups of *cis*-$(NH_3)_2Pt(H\cdot dCMP)_2$ (see Figure 2). These average values are listed in column 6 of Table 2. They follow from equation (8),

$$\Delta\, pK_{a/av} = \frac{1}{2}(\Delta\, pk_1 + \Delta\, pk_2) \quad (8)$$

which is identical with equation (9):

$$\Delta\, pK_{a/av} = pK^{H}_{H(L)} - \frac{1}{2}(pK^{H}_{Pt(H\cdot L)_2} + pK^{H}_{Pt(L)(H\cdot L)}) \quad (9)$$

In equation (9) the difference is taken between the $pK_a$ value of the free ligand and the average of $pK_{a/1}$ and $pK_{a/2}$ for the complex formed with two such ligands by their coordination to *cis*-$(NH_3)_2Pt^{2+}$. This latter method is identical with the one we have applied before.[7-9] In the footnotes to Table 2 some detailed examples for the calculation procedures indicated above are given.

From the $\Delta\, pK_{a/av}$ value in row 1 and column 6 of Table 2 it is evident that the acidifying effect on the two H(N1) sites of $Pt^{2+}$ coordinated to the N7 sites in the guanine residues of *cis*-$(NH_3)_2Pt(9\text{-}EtG)_2^{2+}$ is quite significant ($\Delta\, pK_{a/av}$ = 1.23). The corresponding

**Table 2.** Micro Acidity Constants for *cis*-$(NH_3)_2Pt(L)_2$ Species (defined in analogy to Figure 3) and Extent of the Acidification ($\Delta\, pK_{a/av}$; (eqs (8) and (9))[a] by Nucleobase-Coordinated *cis*-$(NH_3)_2Pt^{2+}$ on H(N1) Sites and $-P(O)_2(OH)^-$ Groups (25°C; $I$ = 0.1 M, $NaNO_3$)

| $H_2(L)$ | $pk^1 = pk_1$ [b] | $pk_2 = pk^2$ [b] | $\Delta\, pk_1$ [c,d] | $\Delta\, pk_2$ [d] | $\Delta\, pK_{a/av}$ [a,e] |
|---|---|---|---|---|---|
| $Pt(9\text{-}EtG)_2^{2+}$ | 8.32 | 8.37 | 1.25[c] | 1.20 | 1.23 |
| $Pt(H\cdot dGMP)_2$ | 5.87 | 5.99 | 0.42[d] | 0.30[d] | 0.36[e] |
| $Pt(dGMP)_2^{2-}$ | 9.03 | 9.18 | 0.53 | 0.38 | 0.46 |
| $Pt(H\cdot dCMP)_2$ | 6.03[b] | 6.17 | 0.21 | 0.07 | 0.14 |

[a] The sites of acidification are for rows 1 and 3 H(N1) and for rows 2 and 4 $-P(O)_2(OH)^-$.

[b] See text in Section 3.3 and Figure 3.

[c] Example for row 1 and column 4:

$\Delta\, pk_1 = pK^{H}_{9\text{-}EtG} - pk_1 = (9.57 \pm 0.04) - (8.32 \pm 0.01) = 1.25 \pm 0.04$

[d] Examples for row 2 and columns 4 and 5:

$\Delta\, pk_1 = pK^{H}_{H(dGMP)} - pk_1 = (6.29 \pm 0.01) - (5.87 \pm 0.03) = 0.42 \pm 0.03$

$\Delta\, pk_2 = pK^{H}_{H(dGMP)} - pk_2 = (6.29 \pm 0.01) - (5.99 \pm 0.02) = 0.30 \pm 0.02$

[e] Example for row 2 and column 6:

$\Delta\, pK_{a/av} = 1/2(\Delta\, pk_1 + \Delta\, pk_2) = 1/2[(0.42 \pm 0.03)+(0.30 \pm 0.02)] = 0.36 \pm 0.04$

$= pK^{H}_{H(dGMP)} - 1/2\, (pK^{H}_{Pt(H\cdot dGMP)_2} + pK^{H}_{Pt(dGMP)(H\cdot dGMP)})$

$= (6.29 \pm 0.01) - 1/2[(5.57 \pm 0.03)+(6.29 \pm 0.02)] = 0.36 \pm 0.04$

effect in *cis*-$(NH_3)_2Pt(dGMP)_2^{2-}$ is considerably lower ($\Delta$ p$K_{a/av}$ = 0.46) which is probably the result of the counterbalance in the charge by the two -$P(O)_3^{2-}$ residues. On the other hand the effect of the N7-coordinated $Pt^{2+}$ on the two -$P(O)_2(OH)^-$ residues in *cis*-$(NH_3)_2Pt(H{\cdot}dGMP)$ is of the same order ($\Delta$ p$K_{a/av}$ = 0.36), which is kind of surprising because in this latter case only a through-space effect can operate; this is different in the case of H(N1) and the N7-coordinated $Pt^{2+}$ ($\Delta$ p$K_{a/av}$ = 0.46) because here both sites are part of the aromatic purine residue. Why the acidifying effect of $Pt^{2+}$ in *cis*-$(NH_3)_2Pt(H{\cdot}dCMP)_2$ ($\Delta$ p$K_{a/av}$ = 0.14 ± 0.03), where it is N3-bound, is by about 0.2 p$K$ units lower than in *cis*-$(NH_3)_2Pt(H{\cdot}dGMP)_2$ ($\Delta$ p$K_{a/av}$ = 0.36 ± 0.04) despite the fact that in both instances the two -$P(O)_2(OH)^-$ groups are acidified, is not clear. Maybe the spatial orientation of the $Pt^{2+}$-coordinated nucleotides is different.

### 4. CONCLUSIONS

It is evident from the present study that a nucleobase-coordinated *cis*-$(NH_3)_2Pt^{2+}$ affects only little the basicity of phosphate residues of nucleoside 5'-monophosphates; the same may be surmised for the phosphate groups in the backbone of DNA. As the basicity of phosphate groups is only slightly lowered, one may assume that the metal ion affinity of these groups is still quite pronounced; in fact, for *cis*-$(NH_3)_2Pt(dCMP)_2^{2-}$ this has already been proven.[9] Consequently, one may suggest that, e.g., $Mg^{2+}$ binding to the phosphate backbone of platinated DNA is not much inhibited by the nucleobase-bound $Pt^{2+}$.

A further interesting observation is the rather significant acidification of the H(N1) sites of guanine residues by N7-coordinated $Pt^{2+}$. This suggests, and evidence pointing into this direction has already been found,[7] that in this way the H(N1) site is transformed into an even better H donor suitable for hydrogen bonding than it is the case in the uncomplexed guanine residue.

### ACKNOWLEDGEMENTS

The competent technical assistance of Mrs. Rita Baumbusch in the preparation of the manuscript is gratefully acknowledged. This study was supported by the Swiss National Science Foundation (H.S.), the 'Deutsche Forschungsgemeinschaft' (B.L.), the 'Fonds der Chemischen Industrie' (B.L.), and the Human Capital and Mobility programme (for B.L. via the Commission of the European Communities in Brussels and for H.S. via the Swiss Federal Office for Education and Science in Berne). This research is also part of the COST D1 programme and received in this context support (H.S.) from the Swiss Federal Office for

Education and Science. B.S. is grateful for a leave of absence from the Zhongshan (Sun Yatsen) University in Guangzhou, People's Republic of China.

### REFERENCES

1. Bloemink, M. J.; Reedijk, J.: *Met. Ions Biol. Syst.* (1996) **32**, 641-685.
2. Whitehead, J. P.; Lippard, S. J.: *Met. Ions Biol. Syst.* (1996) **32**, 687-726.
3. Lippert, B.: *Prog. Inorg. Chem.* (1989) **37**, 1-97.
4. Lippert, B.: *Biometals* (1992) **5**, 195-208.
5. Sigel, H.: *Chem. Soc. Reviews* (1993) **22**, 255-267.
6. Sigel, H.; Song, B.: *Met. Ions Biol. Syst.* (1996) **32**, 135-205.
7. Schröder, G.; Lippert, B.; Sabat, M.; Lock, C. J. L.; Faggiani, R.; Song, B.; Sigel, H.: *J. Chem. Soc. Dalton Trans.* (1995), 3767-3775.
8. Song, B.; Feldmann, G.; Bastian, M.; Lippert, B.; Sigel, H.: *J. Inorg. Biochem.* (1995) **59,** 141.
9. Song, B.; Feldmann, G.; Bastian, M.; Lippert, B.; Sigel, H.: *Inorg. Chim. Acta* (1995) **235**, 99-109.
10. Sigel, H.; Zuberbühler, A. D.; Yamauchi, O.: *Anal. Chim. Acta* (1991) **255**, 63-72.
11. Sigel, H.; Massoud, S. S.; Corfù, N. A.: *J. Am. Chem. Soc.* (1994) **116**, 2958-2971.
12. Sigel, H.; Song, B.; Lippert, B.; et al.: results to be published.
13. Martin, R. B.: *Met. Ions Biol. Syst.* (1979) **9**, 1-39.
14. Sigel, H.; Massoud, S. S.; Tribolet, R.: *J. Am. Chem. Soc.* (1988) **110**, 6857-6865.

**Received: March 23, 1996 - Accepted: April 25, 1996 -**
**Received in revised camera-ready format: April 26, 1996**

